# Metabolomics analysis of serum in a rat heroin self-administration model undergoing reinforcement based on ^1^H-nuclear magnetic resonance spectra

**DOI:** 10.1186/s12868-018-0404-5

**Published:** 2018-03-05

**Authors:** Tingting Ning, Changlong Leng, Lin Chen, Baomiao Ma, Xiaokang Gong

**Affiliations:** 1 College of Life Science, Jianghan University, Sanjiaohu Road, Wuhan, 430056 China; 2 Wuhan Institute of Biomedical Science, Jianghan University, Wuhan, 430056 China

**Keywords:** Heroin self-administration, Metabolomics, ^1^H-nuclear magnetic resonance, Reinforcement

## Abstract

**Background:**

Understanding the process of relapse to abused drugs and ultimately developing treatments that can reduce the incidence of relapse remains the primary goal for the study of substance dependence. Therefore, exploring the metabolite characteristics during the relapse stage is valuable.

**Methods:**

A heroin self-administered rat model was employed, and analysis of the ^1^H-nuclear magnetic resonance-based metabolomics was performed to investigate the characteristic metabolite profile upon reintroduction to the drug after abstinence.

**Results:**

Sixteen metabolites in the serum of rats, including phospholipids, intermediates in TCA (Tricarboxylic Acid Cycle) cycle, keto bodies, and precursors for neurotransmitters, underwent a significant change in the reinstatement stage compared with those in the control group. In particular, energy production was greatly disturbed as evidenced by different aspects such as an increase in glucose and decrease in intermediates of glycolysis and the TCA cycle. The finding that the level of 3-hydroxybutyrate and acetoacetate increased significantly suggested that energy production was activated from fatty acids. The concentration of phenylalanine, glutamine, and choline, the precursors of major neurotransmitters, increased during the reinstatement stage which indicated that an alteration in neurotransmitters in the brain might occur along with the disturbance in substrate supply in the circulatory system.

**Conclusions:**

Heroin reinforcement resulted in impaired energy production via different pathways, including glycolysis, the TCA cycle, keto body metabolism, etc. A disturbance in the substrate supply in the circulatory system may partly explain heroin toxicity in the central nervous system. These findings provide new insight into the mechanism underlying the relapse to heroin use.

**Electronic supplementary material:**

The online version of this article (10.1186/s12868-018-0404-5) contains supplementary material, which is available to authorized users.

## Background

In general, drug addiction is defined as compulsive, out-of-control drug use, despite negative consequence. It is well known to encompass several behavioral stages: intoxication, bingeing, craving and withdrawal [1]. The dependence potential of a drug varies from substance to substance, and from individual to individual. An article in *The Lancet* compared the harm and dependence liability of 20 drugs, using a scale from zero to three for physical dependence, psychological dependence, and pleasure to create a mean score for dependence. Of the 20 drugs, the score for heroin for all three separate parameters was the highest that is, three which is why heroin has been called the most addictive drug [2].

Heroin (diacetylmorphine) is an opioid. Heroin use in the general population increased from 373,000 in 2007 to 914,000 in 2014 worldwide (http://www.samhsa.gov/). While there are varying regional trends in the extent of illicit drug use, the overall global prevalence of drug use is considered to be stable. In China, there were 2.3 million drug users as of the end of 2015, and the percentage of opioid abusers was more than 40% (China National Narcotic Control Committee, Annual review of drug situation in China, 2015).

Although many pharmacological treatments for heroin addiction have been established to ease craving and other physical symptoms, the effectiveness of these treatments has remained obscure [3]. Furthermore, the complicated neurobiological changes accompanying the molecular mechanism leading to drug addiction are not known in detail. Thus, a “global” qualitative and quantitative method will help to provide a complete picture that directly reflects the physiological alterations in the body fluid when person becomes addicted to drugs or is undergoing withdrawal symptoms. Given that the overall health status of an individual is captured by his or her metabolic state, which is a reflection of what has been encoded by the genome and modified by environmental factor, metabolomics has the potential to have a greater impact upon medical practice by providing a wealth of relevant biochemical data compared with that provided by genomics, transcriptomics or proteomics [4].

Metabolomics involves the study of the repertoire of small molecules, or metabolites present in a cell, tissue, organ, or biological fluid. A wide variety of methods have been used to separate and quantify components of the metabolome, and no single analytical platform can capture all metabolomic information in a sample [5]. Liquid and gas chromatography-mass spectrometry, nuclear magnetic resonance (NMR) spectroscopy, and liquid chromatography with electrochemical detection are all used. Overall, NMR is a nondestructive technique and in spite of the overlapping chemical shifts for some metabolites it is generally highly effective for structural elucidation [6].

Animal models involving drug self-administration (SA), conditioned place preference (CPP), and drug discrimination paradigms, have been developed to study specific aspects of drug addiction and substance abuse disorders [7]. Compared with that provided by other models of addiction, SA provides the most direct point-to-point correspondence with addictive behavior that occurs in the natural environment. For this reason, SA has a high degree of face validity [8]. Typically, a drug SA paradigm involves acquisition, extinction or abstinence and reinforcement. Different types of treatments have been found to induce reinforcement, and each of them is clearly analogous to the events that can trigger relapse in human drug abusers. In clinical practice, the risk of relapse remains high even after completion of treatment and prolonged abstinence. Therefore, understanding the vulnerability to relapse is crucial for the development of an effective treatment for drug addiction.

Recently, many studies have applied the metabolomics platform to find molecular markers for drug dependence and some results have been obtained [9–11]. However, none of these studies utilized the SA model, whereas the CPP model has been widely used. In addition, few studies have encompassed the metabolomics data during the reinstatement stage. Although brain tissue and cerebrospinal fluid are obvious samples to use when searching for biomarkers in central nervous system (CNS) disease, the use of serum has some advantages. The most important is that the identification of serum biomarkers more easily translated into clinical use. In addition, there are many pieces of evidence suggesting that changes in the CNS might be correlated with changes in the periphery [12–14]. Thus, in this paper we used ^1^H-NMR-based metabolomics technology to analyze the serum metabolite profiling of rats after reinstatement.

## Results

### Self-administration

**Fig. 1:**
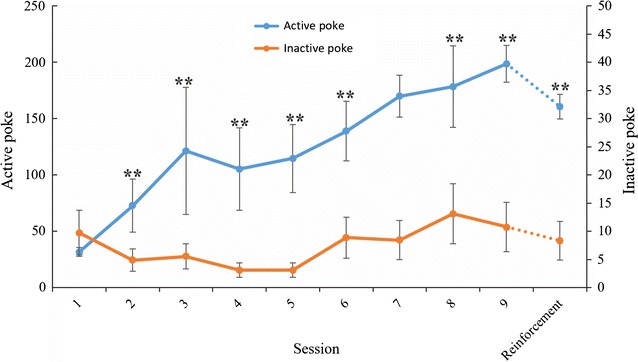
Heroin self-administration. Active and inactive nose pokes during heroin self-administration over 9 consecutive training days and the reinforcement stage (n = 7, the data are expressed by mean ± SEM). Significant difference between the number of active poke and inactive poke in each session was indicated by ** (p < 0.01)

A total of twenty rats were catheterized through the jugular vein, and eight of them were put back into their home cage. The remaining twelve were used for heroin self-administration, but five of them were excluded because they did not meet the criteria for heroin self-administration. Average active and inactive nose poke is shown in Fig. 1. The animals exhibited reliable self-administration of heroin as indicated by the increase in the number of active nose-poke responses (p < 0.05). The drug-induced reinforcement of the self-administered rats was tested after 14 days of abstinence. According to our experience, after 14 days of abstinence, responses of the animals on the active nose poke would decrease gradually, but when re-exposed to drugs, the animals would resume drug-seeking or craving behavior [15, 16]. When the animals were reintroduced to heroin, the average active nose-poke response for the self-administered rats resumed to more than 150, which means that the brain systems involved in the discriminative stimulus properties or the rewarding effect were active. After reinforcement test, serum samples were collected.

## ^1^H-NMR spectra NMR spectra

Representative ^1^H-600 MHz NMR spectra of the serum in a heroin SA rat showing the most representative metabolites identified in this sample are shown in Additional file 1: Fig. S1. Good quality spectra, characterized by the presence of signals with varying degrees of overlap, were obtained for most of the samples. The metabolites observed in the ^1^H-NMR spectra of serum were mainly associated with amino acids (tyrosine, alanine, valine/leucine/isoleucine, histidine, phenylalanine, lysine, glutamate), energy metabolism (α-glucose, succinate, pyruvate), keto body metabolism (acetone, acetoacetate, 3-hydroxybutyrate), and membrane metabolites (phosphocholine, glycerol phosphocholine, lipid).

### Statistical analysis

**Fig. 2:**
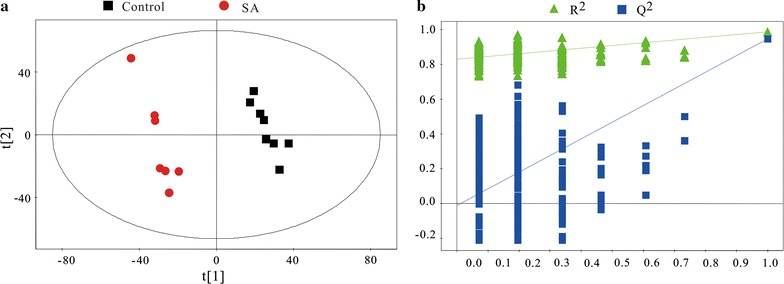
PLS-DA model generated by SIMICA-P software for comparison SA (n = 7) and control group (n = 8) (**a**), R^2^X = 45.4%, R^2^Y = 98.7%, Q^2^ = 0.946; Ellipse was given by Hotteling’s T2 (0.95); Validation plot obtained from 200 permutation tests for the PLS-DA models (**b**), intercept: R^2^ = (0.0, 0.83), Q^2^ = (0.0, − 0.00919). The negative Q^2^ intercept suggested a good predictability of the PLS-DA models

To visualize the clustering of the tested samples, a principal component analysis (PCA) model was constructed. The two first principal components (PCs) showed a distinct separation between the following groups (Additional file 1: Fig. S2). All samples fell in the ellipse representing the Hotelling T2 with 95% confidence. Generally, PC1 explained 28.8% of the total variance, and PC2 explained 17.2%. Better classification and clear separation could be observed in the PLS-DA (partial least squares-discriminate analysis) model (Fig. 2a) with good model parameters (R^2^Xcum = 0.454, Q^2^cum = 0.946). Q^2^ (cum) obtained from the cross-validation for PLS-DA was 0.946, much higher than 0.5, a widely used threshold value for a good multivariate model of metabolomics data. To avoid overfitting, a cross-validation, permutation test was conducted to confirm the validity and predictability of the supervised models (Fig. 2b). The class labels of the tested groups were permuted and randomly assigned to different observations. With the permutated class labels, 200 new supervised models were built. R^2^ and Q^2^ within each model were calculated and a regression line was drawn. The Q^2^ intercept value obtained from the regression line lower than 0.05 was indicative of a valid model. A negative Q^2^ intercept suggested a good predictability of the PLS-DA models.

**Fig. 3:**
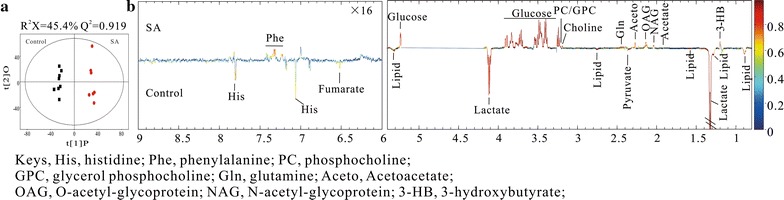
OPLS-DA score plot (**a**) and corresponding coefficient plot (**b**) derived from the ^1^H NMR spectra of extractions of serum obtained from control group (n = 8) and SA group (n = 7). A correlation coefficient of |r| > 0.707 (df = 6) was used as the cut-off value that gave a statistically significant result at the level p < 0.05. The loading maps show the significance of metabolite variations between two classes. Peaks in the positive and negative direction indicate the metabolites that are more abundant in SA group and in the control group respectively

To evaluate the metabolic differences between the two groups, OPLS-DA (orthogonal projections to latent structures discriminant analysis) was performed on the NMR spectral data, and the loading plots displayed significant differences between the two classes. Here, a correlation coefficient of |r| > 0.707 (df = 6) was used as the cut-off value that gave a statistically significant result at the level p < 0.05. In total, 16 of the 27 metabolites were recorded as being significantly different between the SA and control group (Fig. 3). The statistically significant metabolites are listed in Additional file 1: Table S1.

**Fig. 4:**
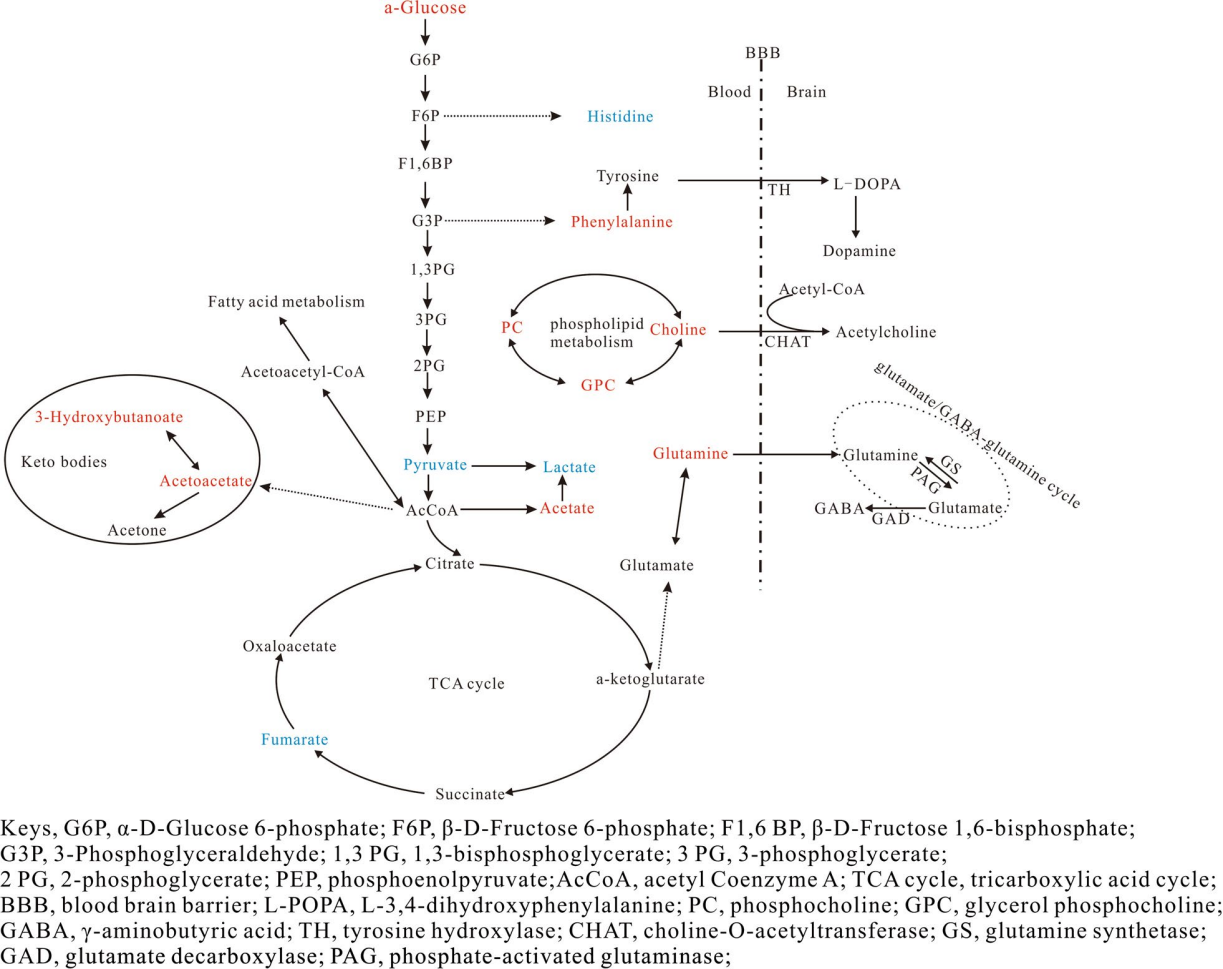
Metabolic reaction networks of characteristic metabolites found differentially expressed in serum between control (n = 8) and SA (n = 7) group. Metabolites in red indicate that the metabolite is in higher concentration in SA group, and metabolites in blue indicate that the metabolite is in lower concentration in SA group

Furthermore, the metabolic pathways derived from the characteristic metabolites were used to assess the major metabolic functions of them. Thirteen changed metabolites were used as the input into the KEGG (Kyoto Encyclopedia of Genes and Genomes, http://www.kegg.jp/), not including NAG (*N*-acetyl-glycoprotein), OAG (*O*-acetyl-glycoprotein) and lipids, since these three terms were loosely defined. As shown in Fig. 4, disordered metabolic reaction pathways are mostly involved in phospholipid metabolism, keto body metabolism and central carbon metabolisms, and three kinds of amino acids were also disturbed. Notably, the relative concentrations of three compounds, choline, glutamine and phenylalanine were elevated. Since these metabolites are the precursors of several important neurotransmitters, i.e., acetylcholine, γ-aminobutyric acid/glutamate and dopamine, we discuss the function of these three metabolites in more detail.

## Discussion

### Self-administration

Relapse is considered a central characteristic of addiction and possibly the most important to overcome. A primary goal of these studies was to understand the human relapse process and, ultimately, to develop treatments that reduce the incidence of relapse. The reinstatement procedure was used to model relapse following a period of abstinence. In this study, reinforcement was induced by re-exposure to the self-administered drug, and the simulation was presumed to be analogous to the true state of a drug-abuser upon incidental exposure to the drug after abstinence. From the number of active-poke responses in Fig. 1, we can see that during the 9 sessions of training, the rats acquired a reliable heroin self-administration response since the active nose-poke response increased gradually and remained relatively stable during the last 3 days. After 14 sessions of abstinence, when the rats were induced by 1 mg/kg heroin, the active nose-poke response recovered to more than one hundred which indicated that the desire for the drug was primed again. We wanted to explore what occurred in the body during this critical status.

### Serum metabolome modification with heroin self-administration

A complete segregation between the control and self-administration group was observed on the score plot of the PCA, PLS-DA, and OPLS-DA models, and different types of validation methods also demonstrated that the model was effective and reliable.

Sixteen out of 27 identified metabolites were significantly altered and five were down-regulated, while eleven were unregulated. Based on the KEGG pathway analysis, three interconnected pathways were disturbed. Glycerol-3-phospho choline, phosphocholine, choline are the main metabolites that participate in phospholipid cycling, and pyruvate and fumarate are key intermediates in glycolysis and the TCA cycle, which is the central pathway for energy production, while 3-HB and acetoacetate are a part of the keto body pathway. Importantly, choline, glutamine, and phenylalanine are precursors of important neurotransmitters.

### Cell membrane

In our results, the concentrations of choline, phosphocholine and glycerol phosphocholine were elevated in the SA group. Changes in fatty acid levels in the blood have been correlated with relapse [17], supporting the hypothesis that blood lipids can serve as biomarkers of cocaine-induced neurological dysfunction. The hypothesis that blood lipids can be related to neurological dysfunction and behavior is further bolstered by a recent finding in Alzheimer’s patients demonstrating that select serum glycerol-phospholipids predicted cognitive impairment over 2–3 years with 90% accuracy [18]. All of these results demonstrate that phospholipid profiling in serum may be a potential indication of cell membrane disruption in barrier function associated with drug-addiction.

### Energy

Neuronal activity is extremely energy demanding, and the brain energy supply requires oxidative metabolism of glucose in mitochondria and demands lactic acid from glycolytic processes. In our results, we observed a disturbance in metabolites including α-glucose, pyruvate, which is involved in glycolysis, fumarate, a key intermediate in the TCA cycle, and lactate, which participates in energy production through a different pathway. Numerous papers have revealed a disturbance in energy metabolism by drug abuse [19, 20]. Zheng et al. reported that intermediates of the TCA were elevated after heroin administration and even after heroin withdrawal for 4 days, indicating an upregulation of the TCA cycle for increased energy metabolism and supply. These data suggest that heroin accelerated energy metabolism and produced more ATP during the acquisition and withdrawal stage [21]. However, during the relapse stage, the concentration of fumarate and lactate also decreased which is consistent with our results. These results imply that the energy supply during the acquisition stage, the withdrawal stage and the relapse stage is different. Therefore, extra attention should be paid to the effective treatment of drug addiction.

Glucose, a primary energy substance for brain metabolism, plays an important role in energy homeostasis. Several studies have been performed to investigate the influence of drug abuse on blood glucose levels [22, 23]. Li et al. reported that nicotine priming induced an increase in glucose in the striatum [23]. The increase in plasma glucose induced by administration of methamphetamine (MA) suggested that administration of MA inhibited the production of energy by glycolysis [24]. Thus, these findings combined with our results have revealed that different types of abused drugs can cause an increase in glucose in the blood or other tissues.

Lactate is deemed to supply alternate energy sources for the brain and could be associated with protective preconditioning. Meanwhile, lactate is metabolized through the TCA, and when compared to glucose, it is equivalent with regard to its access to the TCA in neurons [23]. After a nicotine CPP paradigm, an increase in lactate was found in the NAc [9], while nicotine decreased the level of lactate in the NAc and striatum in another study [23]. The reduced levels of lactate in the urine increased after heroin withdrawal [21]. In our study, the levels of lactate in the serum decreased significantly during the relapse stage. This result together with the increase in glucose and decrease in pyruvate and fumarate in the serum explained the involvement of a different pathway in the disturbance in the energy production by heroin self-administration, which should be paid more attention to develop treatments that reduce the incidence of relapse.

### Keto body

Ketone bodies (3-hydroxybutyrate (3-HB), acetoacetate and acetone) serve as an energy source alternative to glucose for the brain [25]. A change in 3-HB had also been reported to be induced by nicotine and MA administration [9, 24]. In our study, 3-HB and acetoacetate were upregulated in the serum. This result was in accordance with the aforementioned increase in glucose and decrease in pyruvate and fumarate. All of the results together indicated that during the reinstatement stage, heroin administration inhibited energy production by glycolysis and oxidative phosphorylation via the TCA cycle and activated ketogenesis in order to produce energy.

Moreover, we noticed that the body weight of the SA group decreased during the training process and recovered a little during abstinence (data not recorded). Therefore, the reduction in body weight might have contributed to the observed disturbance in energy metabolism. Nevertheless, most of heroin abusers lose weight, similar to the reduction observed in SA models. In the future, more dedicated studies are needed to reveal the relationship between heroin administration and energy metabolism.

### Neurotransmitters

Of the 16 changed metabolites in the serum, three are precursors of neurotransmitters, and their relative concentrations were elevated in the serum of the SA group. Choline, in combination with acetyl-CoA, is catalyzed by the enzyme choline acetyltransferase (CHAT) to produce acetylcholine (ACH). CHAT is the rate-limiting enzyme for the formation of ACH and synaptic levels of ACH are regulated by CHAT. Cholinergic neurons are involved in brain learning and reward function. Studies have shown that acute heroin treatment significantly increases the number of CHAT-positive cells in the nucleus accumbens shell [1], and another study demonstrated that cure-induced reinstatement was inhibited by physostigmine, an inhibitor of acetylcholinesterase, in the NAc [26]. Together, these results could depict a pathway through which the level of choline in the blood regulates the function of cholinergic neurons and then influences heroin relapse behavior.

Phenylalanine is catalyzed to tyrosine by phenylalanine hydroxylase in the liver, and l-tyrosine enters through the blood–brain barrier and is then converted to l-DOPA (l-3,4-dihydroxyphenylalanine), which is subsequently converted into dopamine by the enzyme DOPA decarboxylase. An increase in dopamine transmission in the nucleus accumbens shell is characteristic of drug addiction [27], and dopamine receptor agonists and antagonists have been widely investigated as putative pharmacological therapies for addiction [28]. The rate-limiting enzyme of dopamine formation is tyrosine hydroxylase (TH). Changes in TH levels in the mesolimbic pathway have been related to drug addiction, but different results have been obtained in each of the relevant studies [29–31].

The release of glutamine into the periphery may provide the most important mechanism for the removal of excess nitrogen from the brain. The glutamate-glutamine cycle between astrocytes and neurons is well characterized [32]. Aminobutyric acid (GABA), the chief inhibitory neurotransmitter is synthesized in the brain from glutamate using the enzyme glutamate decarboxylase (GAD). GABA and glutamate are involved in memory, learning and synaptic plasticity, and they are modulated by drugs of abuse [33]. Glutamine not only serves as the precursor of the neurotransmitters but also plays an important role in energy production. The psychotropic effects of heroin on neurotransmitters and the nervous system are well known. Our result that the upregulation of these three metabolites in the serum indicated that a disturbance in the substrate supply in the circulatory system could perhaps partly explain heroin toxicity in the central nervous system. To validate this hypothesis, immunohistochemistry using specific brain tissue should be done to test the expression level of several critical enzymes including CHAT, GAD, and TH. Moreover, as NMR-based metabolomics analysis is more quantitative than qualitative, a fine quantification using a standard should be carried out to confirm the change in the three precursors of the neurotransmitters.

Opioids are known to interfere with normal gastrointestinal (GI) motility by delaying transit, resulting in adverse effects such as bowel dysfunction [34]. These effects are primarily mediated by peripheral μ-opioid receptors, which are widely distributed in the submucosa [35]. The slowed GI motility disturbs the profiling of metabolites in the serum. In addition, heroin administration leads to an alteration in gut microbiota [36], and gut microbiota can in turn impact serum metabolites. Therefore, more well designed studies are needed to verify whether the observed changes in metabolites were caused by heroin reinforcement directly or were an indirect result mediated by gut motility/gut microbiota.

## Conclusions

In the present study, metabolite profiling in the serum of heroin self-administered rats that underwent reinforcement was analyzed using ^1^H NMR-based metabolomics. Our results suggest that heroin reinforcement resulted in impaired energy production via different pathways, including glycolysis, the TCA cycle, and keto body metabolism. The cell membrane metabolism was also disturbed. More importantly, the concentration of choline, phenylalanine and glutamine, the precursors of different dominant neurotransmitters, were upregulated in the blood, and this finding provides new insight into the mechanism through which drug abuse results in toxicity in the central nervous system.

## Methods

### Animals

Male Sprague–Dawley rats (260 ± 20 g) obtained from the Vital River Laboratory Animal Technology Co. Ltd (SCXK-2012-001) were housed individually in home cages in a temperature-controlled ventilated room with a reversed 12-h light/dark cycle (lights onset 08:00 p.m., offset 08:00 a.m.). Food and water were freely available. All procedures involving animals and their care were carried out according to the governmental guidelines on animal experimentation, National Institutes of Health “Principles of Laboratory Animal Care” and the Guide for the Care and Use of Laboratory Animals. The experiment was approved by Jianghan university experimental animal ethical review committee and the approval number is 201608002.

### Chemicals and drugs

Diacetylmorphine hydrochloride (heroin) was obtained from the Hubei Public Security Bureau and was dissolved in 0.9% NaCl.

### Surgery

The rats (n = 20) received catheter surgery under anesthetization with sodium pentobarbital (50 mg/kg, i.p.). A silicon catheter (3.5 cm in length, 0.58 mm inner diameter, 0.91 mm outer diameter; BPU-30, Instech, Plymouth Meeting, PA, USA) was implanted and secured within the right jugular vein. The other end of the catheter (10 cm, PE20) was passed subcutaneously to the back and exited into a fluid-connector. Following surgery, each rat was housed individually in its cage and received a daily intravenous infusion of gentamicin (0.16 mg/kg) followed by 0.2 ml of a heparinized (1%) sterile saline solution to maintain catheter patency.

### Self-administration

After 1 week of recovery, twelve rats were trained to self-administer heroin during daily 4-h sessions under a fixed ratio 1 schedule of reinforcement until stable responses occurred. Rats received a single heroin infusion (0.06 mg/kg) following an active nose-poke response. Each infusion was paired with a 5 s illumination of a light in combination with the noise of the infusion pump. Following each infusion, a time-out period was imposed for 20 s, during which responding was recorded but had no programmed consequences. After the timeout period, the red house light once again turned on, and a new self-administration training session started. Rats were put pack into their individual home cages shortly after the session. The rats continued SA training until stable responses occurred. The responses were considered stable when two criteria were met: 1) the number of active nose-poke responses was significantly more than the number of inactive nose-poke responses (p < 0.05); 2) less than 10% variability was obtained in the number of active nose–poke responses for at least three consecutive days. Rats meeting the acquisition criterion entered into a spontaneous abstinence session. All animals were kept in their home cage during the 14 days of abstinence. Then, drug-induced reinforcement was tested. All rats were injected with 1 mg/kg heroin (i.p.) and then placed into the operant chamber for 4 h, during which time the nose pokes had no programmed consequences, but the number of nose-poke responses were recorded. After the reinforcement test, blood was collected from the orbital sinus under chloral hydrate anesthesia. Serum samples were prepared and frozen in liquid nitrogen immediately after collection and stored at − 80 °C until analysis. The remaining eight rats comprised the control group and remained in their home cage after surgery throughout the operant process, and serum samples were collected at the same time and in the same way as for the SA group.

## ^1^H NMR spectroscopy of serum

Prior to the metabolomics experiment, the serum samples were thawed at room temperature and vortexed. Next, mixtures of 200 μL of serum and 400 μL of saline solution (prepared from 0.9% NaCl, 15% D_2_O and 3 mM TSP) were mixed again. After centrifugation (10 min at 120,00*g*) an aliquot of 550 μL of each sample supernatant was subsequently transferred to a 5 mm NMR tube. The samples were maintained at 4 °C before measurement.

## ^1^H-NMR measurement

The NMR spectra of serum samples were recorded at 298 K using an Agilent Direct Drive2-600 MHz spectrometer (Agilent, USA) operating at a proton frequency of 599.83 MHz. The NMR spectra of the serum samples were recorded using a 1D CPMG pulse sequence with water presaturation in the Agilent notation. For each sample, 128 scans were collected with a spin-echo delay of 350 μs, 100 loops, relaxation delay of 2 s, acquisition time of 2.30 s, TD of 64 k, and SW of 16 ppm. Spectra were processed with a line broadening of 1 Hz and manually phased and baseline corrected using MestReNova 7.0 software (Mestrelab Research SL) and referenced to the α-glucose signal, δ = 5.23 ppm. The segments of δ 4.18–5.22 ppm and 5.4–6.0 ppm in spectra were removed to exclude the influence of the residual water and urea resonance. Each ^1^H-NMR spectrum over the range of δ 0.8–9.0 was integrated into an equal width (0.002 ppm). Each bucketed region was then normalized to the total sum of the spectral integrals to compensate for overall concentration differences.

### Statistical data analysis

Prior to chemometrics analysis, the data set was Ctr scaled. For primary visualization, distribution and clustering, the principal component analysis (PCA) model was applied using SIMICA-P (V11.0, Umetrics AB, Umea, Sweden). Next, a PLS-DA with a sevenfold cross validation procedure (CV, 1/7 of the samples being excluded from calculations in each round) to determine the variation between data sets was adopted.

The concentration of the metabolites based on ^1^H NMR spectroscopy was calculated as the relative signal integrals of the non-overlapping resonances. The metabolite resonances were identified according to assignments published in the literature and in on-line databases (Human Metabolome Data Base, http://www.hmdb.ca/). The percentage difference was calculated based on the average values of relative signal integrals in each group. The OPLS-DA method was utilized to identify the differential metabolites between the two groups on Ctr-scaled data. Here, a correlation coefficient of |r| > 0.707 (df = 6) was used as the cut-off value that gave a statistically significant result at the level p < 0.05. To identify the most altered metabolic pathways, a set of significantly altered metabolites was used as the input for the KEGG Pathway Analysis.

## Additional file

**Additional file 1: Figure S1.** ^1^H NMR spectra. **Figure S2.** PCA model. **Table S1.** Correlation coefficients.
