# Supplementary material for: Metabolomics analysis of serum in a rat heroin self-administration model undergoing reinforcement based on 1H-nuclear magnetic resonance spectra
Source: BMC Neurosci. 2018 Mar 5;19:4. doi: 10.1186/s12868-018-0404-5 (PMC5836429; doi:10.1186/s12868-018-0404-5)
Supplement: Supplementary file 1 — Additional file 1: Figure S1. 1H NMR spectra. Figure S2. PCA model. Table S1. Correlation coefficients. [file 12868_2018_404_MOESM1_ESM.pptx]

## Slide 1
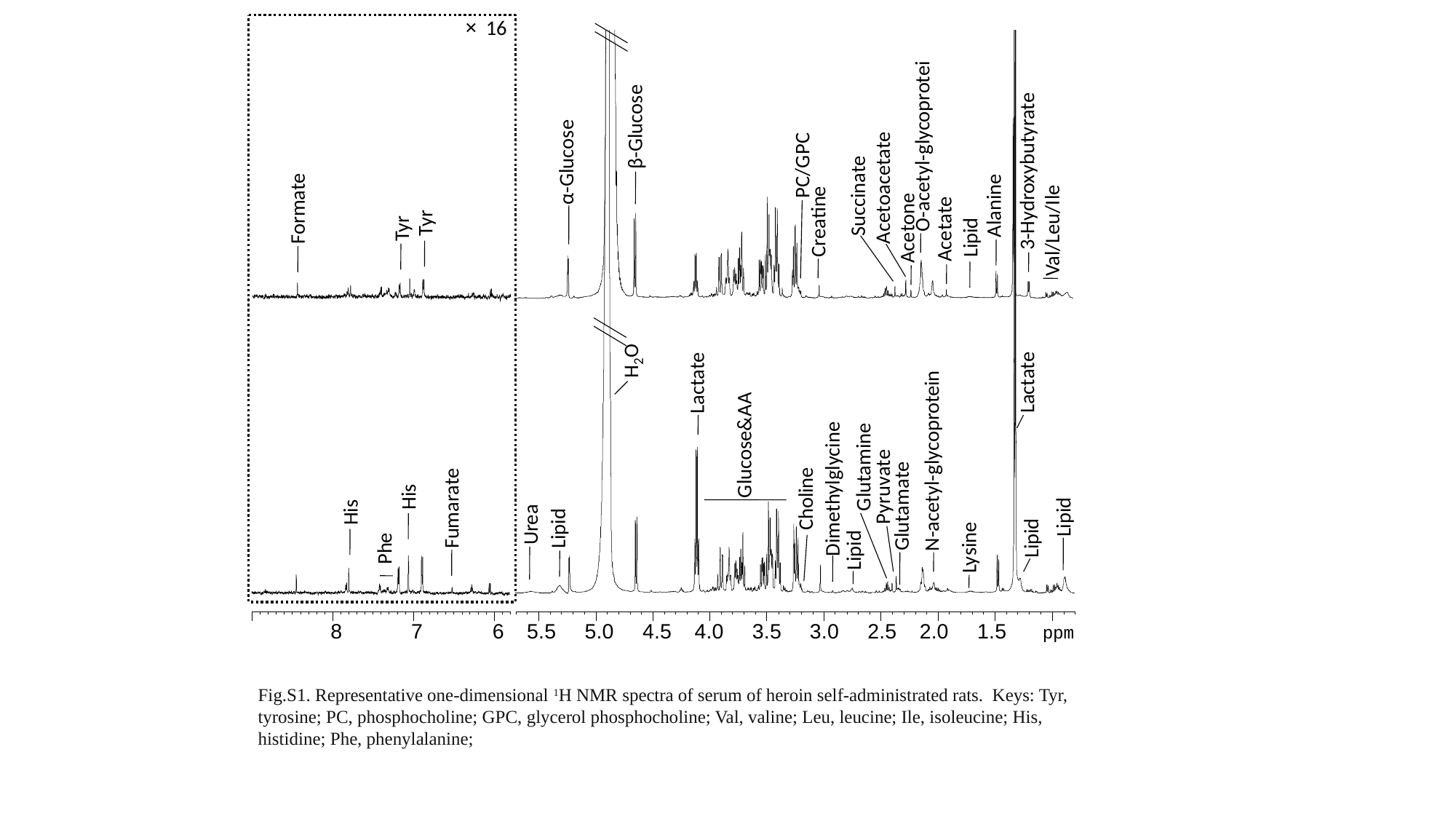

Fig.S1. Representative one-dimensional 1H NMR spectra of serum of heroin self-administrated rats. Keys: Tyr, tyrosine; PC, phosphocholine; GPC, glycerol phosphocholine; Val, valine; Leu, leucine; Ile, isoleucine; His, histidine; Phe, phenylalanine;

## Slide 2
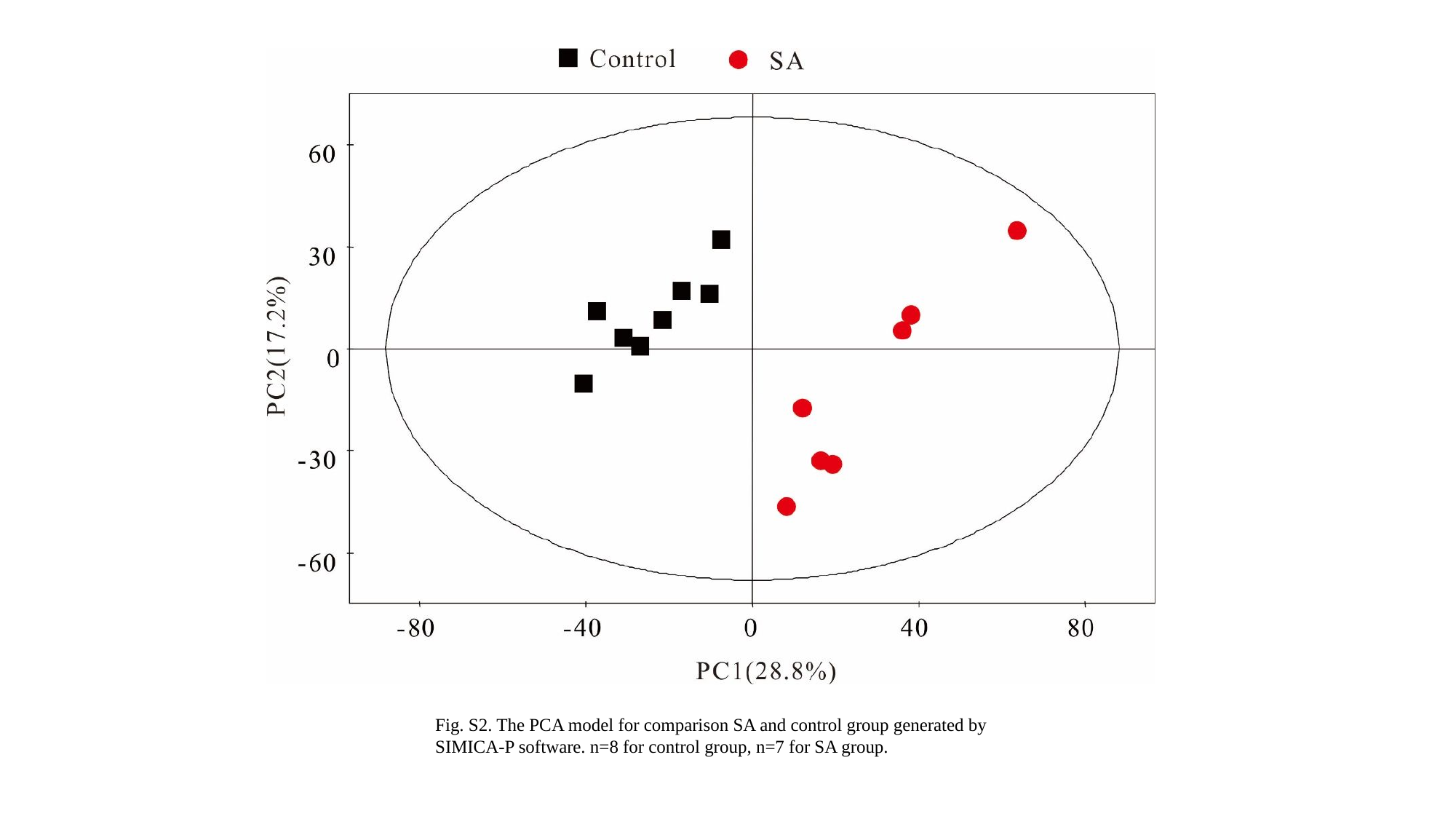

Fig. S2. The PCA model for comparison SA and control group generated by SIMICA-P software. n=8 for control group, n=7 for SA group.

## Slide 3
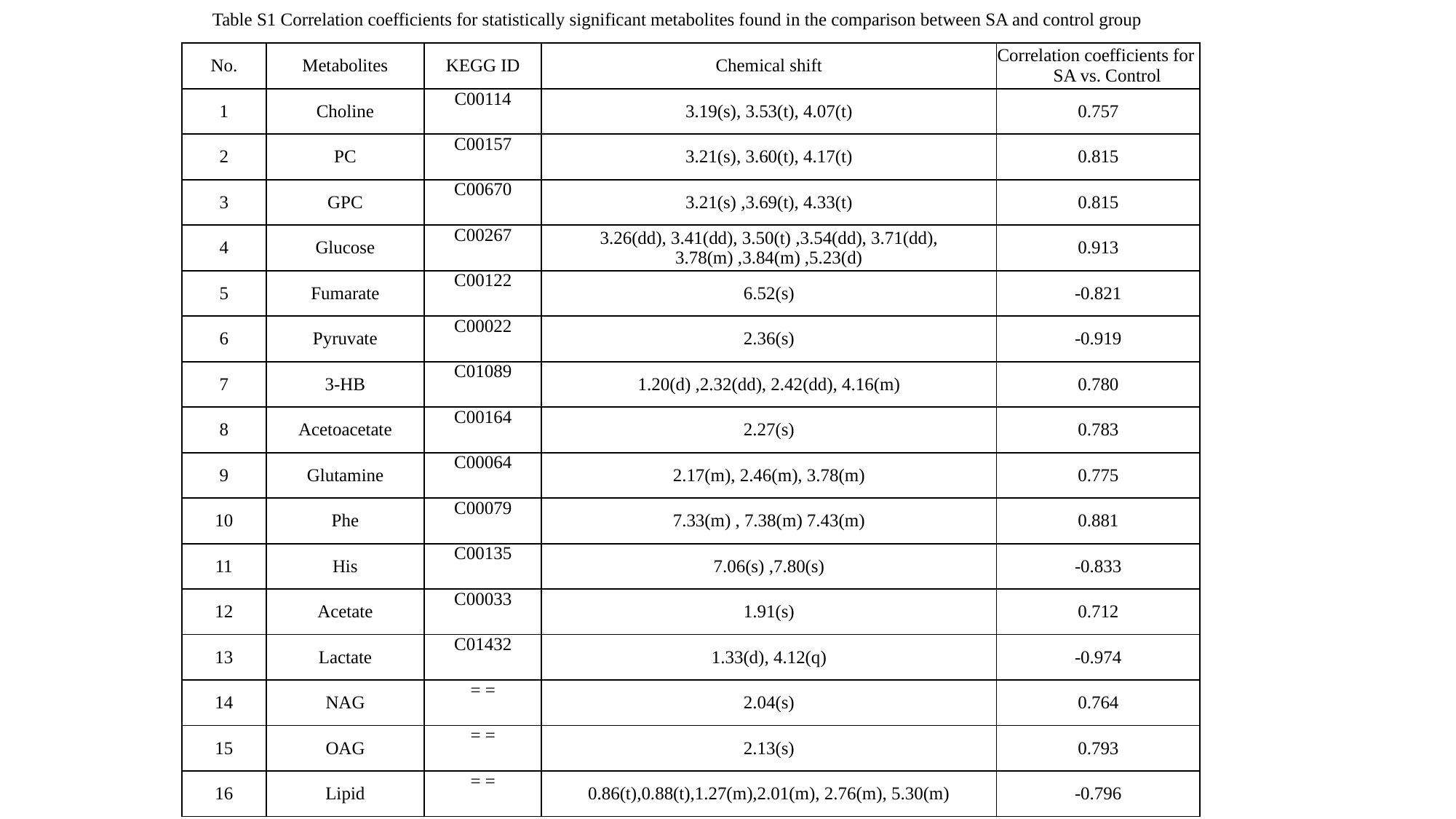

Table S1 Correlation coefficients for statistically significant metabolites found in the comparison between SA and control group
| No. | Metabolites | KEGG ID | Chemical shift | Correlation coefficients for SA vs. Control |
| --- | --- | --- | --- | --- |
| 1 | Choline | C00114 | 3.19(s), 3.53(t), 4.07(t) | 0.757 |
| 2 | PC | C00157 | 3.21(s), 3.60(t), 4.17(t) | 0.815 |
| 3 | GPC | C00670 | 3.21(s) ,3.69(t), 4.33(t) | 0.815 |
| 4 | Glucose | C00267 | 3.26(dd), 3.41(dd), 3.50(t) ,3.54(dd), 3.71(dd), 3.78(m) ,3.84(m) ,5.23(d) | 0.913 |
| 5 | Fumarate | C00122 | 6.52(s) | -0.821 |
| 6 | Pyruvate | C00022 | 2.36(s) | -0.919 |
| 7 | 3-HB | C01089 | 1.20(d) ,2.32(dd), 2.42(dd), 4.16(m) | 0.780 |
| 8 | Acetoacetate | C00164 | 2.27(s) | 0.783 |
| 9 | Glutamine | C00064 | 2.17(m), 2.46(m), 3.78(m) | 0.775 |
| 10 | Phe | C00079 | 7.33(m) , 7.38(m) 7.43(m) | 0.881 |
| 11 | His | C00135 | 7.06(s) ,7.80(s) | -0.833 |
| 12 | Acetate | C00033 | 1.91(s) | 0.712 |
| 13 | Lactate | C01432 | 1.33(d), 4.12(q) | -0.974 |
| 14 | NAG | = = | 2.04(s) | 0.764 |
| 15 | OAG | = = | 2.13(s) | 0.793 |
| 16 | Lipid | = = | 0.86(t),0.88(t),1.27(m),2.01(m), 2.76(m), 5.30(m) | -0.796 |
